# Transcriptome Fingerprinting Analysis: An Approach to Explore Gene Expression Patterns in Marine Microbial Communities

**DOI:** 10.1371/journal.pone.0022950

**Published:** 2011-08-09

**Authors:** Montserrat Coll-Lladó, Silvia G. Acinas, Cristina Pujades, Carlos Pedrós-Alió

**Affiliations:** 1 Department of Marine Biology and Oceanography, Institut de Ciències del Mar (CSIC), Barcelona, Spain; 2 Departament de Ciències Experimentals i de la Salut, Universitat Pompeu Fabra, Parc de Recerca Biomèdica de Barcelona, Barcelona, Spain; Argonne National Laboratory, United States of America

## Abstract

Microbial transcriptomics are providing new insights into the functional processes of microbial communities. However, analysis of each sample is still expensive and time consuming. A rapid and low cost method that would allow the identification of the most interesting samples for posterior in-depth metatranscriptomics analysis would be extremely useful. Here we present Transcriptome Fingerprinting Analysis (TFA) as an approach to fulfill this objective in microbial ecology studies. We have adapted the differential display technique for mRNA fingerprinting based on the PCR amplification of expressed transcripts to interrogate natural microbial eukaryotic communities. Unlike other techniques, TFA does not require prior knowledge of the mRNA sequences to be detected. We have used a set of arbitrary primers coupled with a fluorescence labeled primer targeting the poly(A) tail of the eukaryotic mRNA, with further detection of the resulting labeled cDNA products in an automated genetic analyzer. The output represented by electropherogram peak patterns allowed the comparison of a set of genes expressed at the time of sampling. TFA has been optimized by testing the sensitivity of the method for different initial RNA amounts, and the repeatability of the gene expression patterns with increasing time after sampling both with cultures and environmental samples. Results show that TFA is a promising approach to explore the dynamics of gene expression patterns in microbial communities.

## Introduction

Information about dynamics of the genes expressed by microbial communities is being explored by several approaches. Expression of specific genes can be successfully determined through quantitative RT-PCR, and microarrays are helpful tools to detect the expression level of a set of known genes. In addition, the 454 pyrosequencing technology has been recently applied to analyze marine microbial metatranscriptomes [1]–[6]. These metatranscriptomics studies of marine microbial communities are very powerful at uncovering active metabolisms and functional processes. However, this technology is still very costly and cannot be applied to a large set of samples. Thus, for example, Hewson et al. [7] analyzed the metatranscriptome of only eight samples: one from station Aloha, four from the Atlantic and three from the Pacific Ocean. These are only eight isolated stations from two huge oceans. If a fingerprinting method had been available, it would have been possible to determine how representative these samples were of the different water masses studied. Therefore alternative high-throughput approaches are needed to systematically compare and detect gene expression profiles with reasonable time and money costs.

Fingerprinting DNA techniques such DGGE [8], [9], RFLP [10], t-RFLP [11] or ARISA [12], [13] are widely used to compare microbial community composition among different samples. These techniques target the predominant taxa and allow the comparison of an extensive number of samples at a relatively low cost. Thus, studies of the seasonal and spatial distribution of both eukaryotes and prokaryotes have been successfully conducted and a fairly robust view of microbial distribution in the oceans has been obtained [9], [14]–[20]. The next step would be to explore how the activity patterns of such communities change and whether they do so in correlation with taxonomic composition or not. A technique equivalent to DNA fingerprinting, however, is not currently available for patterns of gene expression in microbial communities. We developed an approach that has the advantages of fingerprinting, namely it is relatively cheap and allows processing of a large number of samples.

**Figure 1:**
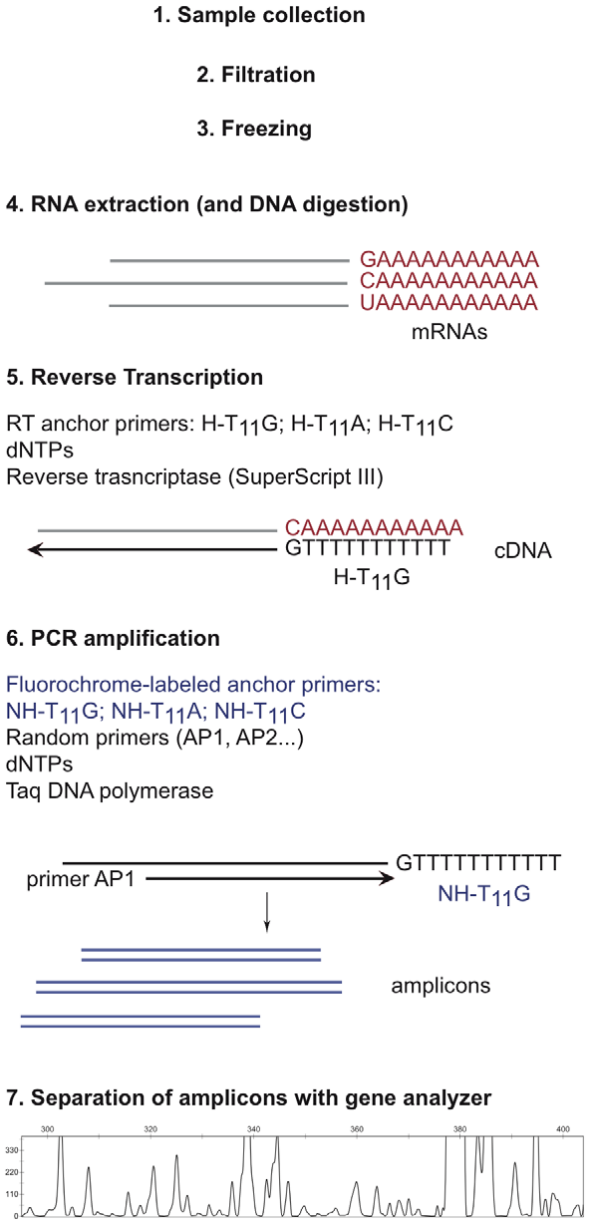
Scheme of the different steps in Transcriptome Fingerprinting Analysis (TFA). Note that in step 4 there is a mixture of ribosomal, transfer, and messenger RNAs. By using primers against the poly(A) tail, step 5 reverse-transcribe mRNAs only.

Here we present an approach to detect gene expression patterns in picoeukaryotic marine microbial communities. Transcriptome Fingerprinting Analysis (TFA) is based on the well-known differential display approach [21], [22], but with some modifications to adapt it to marine microbial ecology studies (Figure 1). In this procedure, nucleic acids are extracted from the natural sample and treated with DNAase to leave only RNA. Then, reverse transcription is carried out with anchor primers. In our case, these primers target the poly(A) tail of eukaryotic mRNAs, insuring that rRNA will not be reverse-transcribed. Next, PCR is carried out with the same anchor primers plus a set of random primers. We used fluorochrome labeled anchor primers for this amplification so that the amplicons could be separated in a conventional gene analyzer. In the end, for each sample we had a profile in which every peak corresponded to an expressed gene. The differences between the expression profiles in two different environments could then be easily explored. Allegedly, each sample should show peaks that were unique to that environment and peaks that were common for a specific set of conditions. The differences are presumably the result of different parameters associated with the specific environment. We determined the sensitivity and repeatability of the method using both cultures of the prasinophyte *Micromonas pusilla*, and natural marine picoeukaryotic communities from the Mediterranean Sea.

## Materials and Methods

### Sampling and collection of biomass

Sea surface samples were obtained from the tip of the Gas pier in the Barceloneta beach (Barcelona) in 8 liter carboys. In experiment 1 (see Table 1), carried out on October 3, 2007, samples were kept on ice and in experiment 2 (Table 1), carried out on September 25, 2008, samples were either kept on ice (ICE samples) or at room temperature (RT samples) until the end of the filtration process. Water was prefiltered through a 200-µm mesh net. Additional water samples were collected during the MODIVUS cruise (17–27 September 2007) on board *R/V García del Cid* at three stations from coastal to open sea. Seawater (8 liters) was collected using Niskin bottles and was also prefiltered through 200-µm mesh net. A piece of 20-µm Nylon mesh was attached to the entrance tube cap of the filtration system and all environmental water samples were filtered first through a 3-µm pore-size polycarbonate filter (Poretics) and then through a 0.2-µm polycarbonate filter (Poretics) using a peristaltic pump (MasterFlex 7553-89 with cartridges Easy Load II 77200-62, Cole-Parmer Instrument Company) to collect the bacteria and picoeukaryotes. Filters were flash-frozen in liquid nitrogen and then stored at −80°C until processed. Total RNA was extracted from the 0.2-µm polycarbonate filters.

**Table 1:** Key to the different experiments showing the sample used and the variables tested in each case.

Experiment	Sample	Variables tested
1	Natural sample	Primer combinations, time since sampling
2	Natural sample	Time since sampling at two temperatures
3	*Micromonas*	Time elapsed at two temperatures
4	*Micromonas*	Light and dark conditions

### *Micromonas* experiments

Axenic cultures of the prasinophyte *Micromonas pusilla* CCMP 1545 obtained from the Provasoli–Guillard National Center for Culture of Marine Phytoplankton (CCMP; https://ccmp.bigelow.org/) were grown in f/2 medium [23] at 19°C under a daily regime consisting of 12 h of light and 12 h of darkness. Growth of *M. pusilla* was followed by flow cytometry (procedure described in [24]) to be sure that the cultures were in exponential growth phase. Experiment 3 (Table 1) was initiated when cultures reached sufficient biomass after 6 days of growth and triplicates of a time zero control were taken. Half of the bottles were kept at 4°C and half at 19°C. After temperature equilibration (about five minutes) all bottles were transferred to the dark. 4°C and 19°C cultures were sampled in triplicate after 15 min, 30 min, 1 h, 2 h and 4 h of incubation. At each sampling point 10 ml of culture were filtered through 0.8-µm-pore-size Durapore Filters and the filters were flash-frozen in liquid nitrogen and kept at −80° until RNA extraction. In experiment 4 (Table 1), a *M. pusilla* culture was growing at 19°C under a 12 h light/12 h dark cycle also until late exponential phase. Then, part of the culture was incubated separately in the dark for 24 hours while the other remained under the light/dark regime. *M. pusilla* cultures were sampled in triplicate under light and dark conditions 24 hours after splitting conditions.

RNA extraction and purification. The procedure was adapted from [25]. For RNA extraction, filters were transferred to 2 ml screw-cap microcentrifuge tubes containing 200-µl of 0.1-mm-diameter zirconia-silica beads (BioSpec Products, Inc.) and 100-µl of 0.5 mm glass beads (BioSpec Products, Inc.) mixed with 450-µl RLT lysis buffer (provided by the RNeasy® Mini Kit Qiagen, Inc.) plus β-mercaptoethanol (Fluka). Samples were mechanically disrupted in a Mini-beadbeater-8TM cell disrupter (BioSpec Products, Inc., Bartlesville, OK) for 10 min. After disruption, samples were incubated on ice for 5 min and the beads were allowed to settle out of the lysis mixture. Samples underwent centrifugation (in an Eppendorf centrifuge at 2100 rcf 1 min). The lysate was transferred to a new tube. 300 µl of lysis solution was added to the vials with beads to increase the final yield. The tubes were shaken vigorously and the supernatant was also recovered. The same volume of 70% ethanol was added to the lysate and samples were purified according to the RNeasy® Mini Kit (Qiagen, Inc.). The isolated total RNA was treated with TurboDNase I (Ambion) to remove contaminating genomic DNA according to the manufacturer's instructions. RNA was aliquoted and quantified by absorbance at 260 nm with a NanoDrop 1000 (Thermo Fisher Scientific Inc., Wilmington, DE).

Reverse transcription and PCR amplification. First-strand cDNA synthesis was conducted with 20 or 40 ng of total RNA as starting material. mRNAs were reverse-transcribed to single-stranded complementary DNA using the SuperScript III reverse transcriptase (Invitrogen) and three different primers, H-T11G (5′-AAGCTTTTTTTTTTTG-3′), H-T11A (5′-AAGCTTTTTTTTTTTA-3′) and H-T11C (5′-AAGCTTTTTTTTTTTC-3′). To denature any secondary structure, an aliquot of each of the RNA extracts plus the oligo(dT) primer were heated for 5 min at 65°C and immediately placed on ice before mixing with the final reaction solution (20 µl): 5× First-Strand buffer, 0.1 M DTT, 40 U RNaseOUT, 10 mM dNTPs and 200 U SuperScript III RT. Reaction mixtures were incubated at 50°C during 50 min, and inactivated by heating 70°C for 15 min. 2 µl of the RT reaction product was used in a subsequent PCR. PCR reactions were carried out using Taq polymerase (Qiagen) in a final reaction volume of 20 µl. Arbitrary primers coupled with the same primers used in the RT reaction but labeled with a fluorescence tag (NED) were used for the amplification of cDNA. Primers targeting the poly(A) tail and arbitrary primers for PCR were from RNAspectra Yellow kit 1 of GenHunter Corporation. These primer sequences are given in Table 2. The PCR cycle was 40 cycles of 94°C for 30 s, annealing at 40°C for 2 min, 72°C for 60 s followed by 1 cycle of 72°C for 5 min in a Techne thermal cycler (Techne, Ltd., Cambridge). A negative control was run for each primer combination to assess the background levels (usually very low, below 20 relative fluorescence units -rfu-) and to ensure that there was no amplification of genomic DNA an aliquot of the RNA extracts was added directly to the PCR.

**Table 2:** Primers used in the present study (from a commercial primer kit: RNAspectra kit of GenHunter).

Primer	Sequence (5′-3′)
RT anchor primers	
H-T_11_G	5′-AAGCTTTTTTTTTTTG-3′
H-T_11_A	5′-AAGCTTTTTTTTTTTA-3′
H-T_11_C	5′-AAGCTTTTTTTTTTTC-3′
Fluorescently-labeled PCR anchor primers	
NH-T_11_G	5′-AAGCTTTTTTTTTTTG-3′
NH-T_11_A	5′-AAGCTTTTTTTTTTTA-3′
NH-T_11_C	5′-AAGCTTTTTTTTTTTC-3′
Arbitrary primers	
H-AP1	5′-AAGCTTGATTGCC-3′
H-AP2	5′-AAGCTTCGACTGT-3′
H-AP3	5′-AAGCTTTGGTCAG-3′
H-AP4	5′-AAGCTTCTCAACG-3′
H-AP5	5′-AAGCTTAGTAGGC-3′
H-AP6	5′-AAGCTTGCACCAT-3′
H-AP7	5′-AAGCTTAACGAGG-3′
H-AP8	5′-AAGCTTTTACCGC-3′

One anchor primer was used in the RT reaction, and the same primer but fluorescently-labeled was combined with one of the arbitrary primers in the subsequent PCR.

### Detection and analysis of peaks

1 µl of PCR product from each sample was mixed with 9 µl of Hi-Di Formamide (Applied Biosystems). 0.5 µl of size standard (ROX 500, Applied Biosystems) was added to every reaction to define the standard curve between 25 and 500 bp. The cDNA peaks obtained ranged in length from 30 to 500 bp, according to the internal size standard used. The mixtures were run on an ABI automated sequencer operating as a fragment analyzer (ABI 3130XL). The sequencer electropherograms were then analyzed using the GeneMarker software, version 1.90 (SoftGenetics, LLC). Raw data were treated with some filters activated according to GeneMarker instructions: baseline substraction, spike removal, auto pull up removal, smooth. The cubic spline algorithm was used to calculate bp lengths of identified fluorescence peaks. The following peak detection thresholds were applied: I) An intensity cutoff of 150 rfu was chosen, although the use of this cutoff may have reduced the diversity of the communities (some peaks larger than background were present below that cutoff. II) The stutter peak filter with a 5% left and right percentage and a peak score between 5 and 7. In addition, the peaks were visually inspected for sporadic inconsistencies in the binning, basically for those peaks with high intensity (larger than 500 rfu) that could make a big difference in the community pattern. Once the peaks were selected, peak areas were used as output from GeneMarker software and were transferred to Microsoft Excel (Seattle, WA) for subsequent analysis.

### Statistical analysis

The peak area data from GeneMarker were standardized (the relative peak heights within a profile were calculated by dividing the height of an individual peak by the total peak height -sum of the heights of all peaks in a pattern-). TFA was evaluated by comparing the number and area of peaks (bands) between electropherograms (profiles). The similarity of TFA profiles derived from different communities was assessed computing Bray-Curtis distances to construct the similarity matrices. Patterns were explored using nonmetric multidimensional scaling (NMDS) and clustering analysis. Primer-E version 6 was used for these analyses [26].

## Results

### Sensitivity and repeatability of TFA

**Figure 2:**
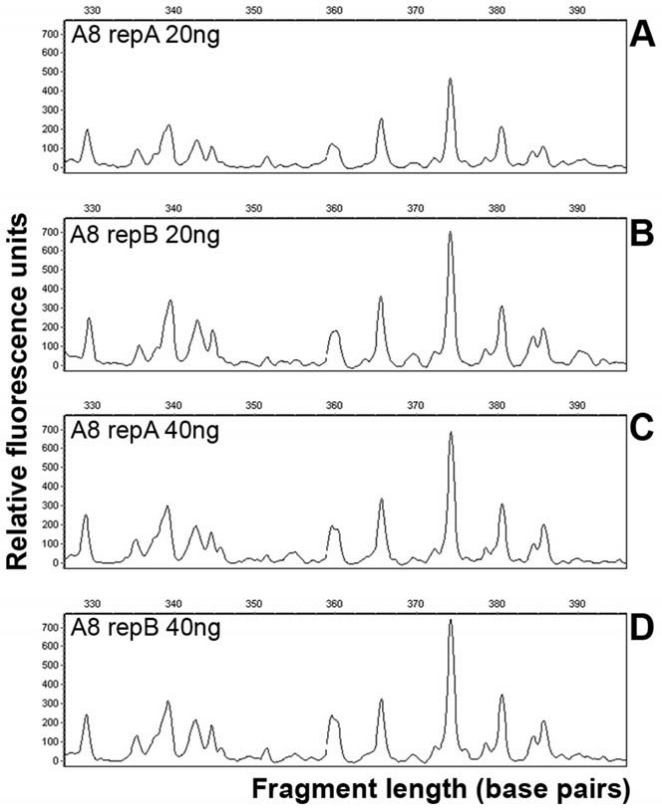
Examples of TFA profiles, showing the sensitivity and repeatability of the technique. All panels are replicates of the same environmental sample. Panels A and B show replicate fingerprints obtained from 20 ng of total RNA and panels C and D from 40 ng of RNA. The horizontal scale goes from 330 to 390 bp from left to right.

The amount of total RNA usually obtained from 8 L of seawater from oligotrophic Blanes Bay was approximately 90 ng. Experiments showed that around 10–20 ng were optimal for good quality patterns. Higher RNA concentrations, such as 80 ng, resulted in lower signal (data not shown). Although TFA was found to be very sensitive, it failed to produce repeatable patterns under the initially assayed conditions with the RNAspectra Yellow Kit 1 (GeneHunter). This kit is based on the MMLV reverse transcriptase (operating at 37°C). Different reverse transcription enzymes were tested and the SuperScript-III enzyme (Invitrogen) produced the desired results. This is an engineered version of the former enzyme active at 50°C. With this enzyme the repeatability was very high, as evidenced by the fact that the electropherograms representing four replicates were identical, both with 20 and 40 ng of RNA (Figure 2). Finally, different times for the reverse transcription reaction were tested and no differences were found between 30 and 60 min (data not shown).

### Elapsed time between sampling and filtration

**Figure 3:**
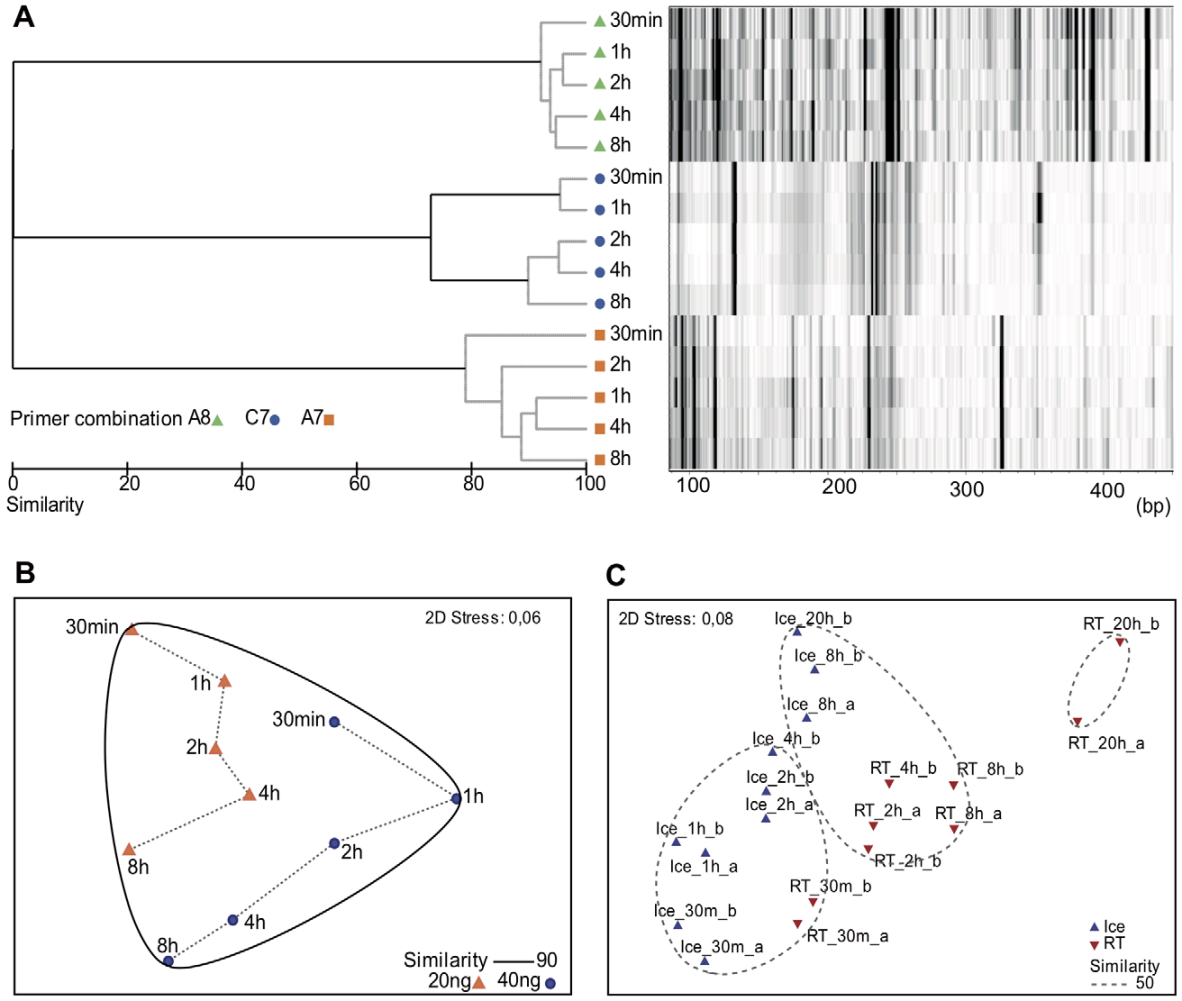
Comparison of TFA profiles from environmental samples filtered at progressively longer times after collection. The shortest practical time was 30 minutes. A) and B) experiment carried out on October 3, 2007 with samples kept on ice; and C) timing experiment carried out on September 25, 2008. A) Cluster analysis from a Bray-Curtis similarity matrix of TFA done with three different primer combinations (A8, C7, and A7) from the same sample filtered at different times between 30 min and 8 h. Gray lines indicate differences were not significant at the 5% level. The lane next to each sample corresponds to the peak pattern (in a base pairs scale) of each sample in a virtual gel. B) NMDS diagram comparing fingerprints obtained using two initial RNA amounts with the A8 primer combination. C) NMDS comparison of samples kept on ice or at room temperature filtered at different times between 30 min and 20 h. Patterns were obtained with the A8 primer combination. Missing replicates are due to low quality electropherograms.

Three different experiments were carried out to test the changes in expression patterns with time elapsed since sampling. Experiments 1 and 2 were done with seawater samples and experiment 3 with a culture of *Micromonas pusilla*. The shortest feasible time between sampling and filtration in the experiments with natural seawater was 30 minutes. The volume filtered was 2–4 L of water in 15–30 min for each time point for all the samples. Water was kept on ice until filtration was completed. The dendrogram in Figure 3A corresponds to experiment 1, carried out on 3 October 2007 in which the samples were filtered 30 min, 1 h, 2 h, 4 h, and 8 h after collection. The cDNA was amplified with three different arbitrary primer combinations (A8, C7 and A7). The patterns obtained with different primer combinations differed substantially both in the identity and quantity of genes retrieved as could be expected (see virtual gel in Figure 3A). Lines in gray indicate that the differences between branches were not significantly different at the 5% level. Slight differences between samples filtered at 30 min and one hour and the rest of the samples were observed with one of the primer combinations (C7).

Samples treated with the primer combination A8 were run with two different initial amounts of RNA (20 and 40 ng). The similarities among the treatments were explored with a NMDS diagram (Figure 3B). A gradual change in the patterns from 30 min to 8 h was observed in both sets of samples. The amount of RNA had a small influence on the resulting pattern. However, all the samples showed a similarity higher than 90% among them. In conclusion, keeping the samples on ice was enough to guarantee that profiles did not differ significantly, even after 8 hours, with two of the three primer combinations tested.

In experiment 2 the effect of keeping samples either on ice or at room temperature was tested (Figure 3C). Duplicates were done for each time point and only one set of primers was used (A8). As expected, samples kept at room temperature during 20 hours differed the most from the initial samples. Samples kept on ice were more similar to the initial ones than their room temperature counterparts for the same sampling times.

**Figure 4:**
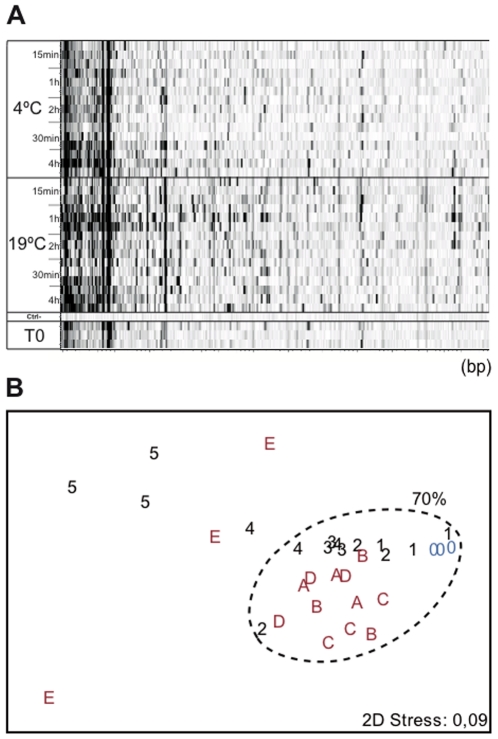
Elapsed time between sampling and filtration in a *Micromonas pusilla* culture. A) Virtual gel of *Micromonas pusilla* fingerprints of triplicate samples filtered at different times between 15 min and 4 h after splitting conditions: 4°C or 19°C samples. Patterns obtained with the A8 primer combination. The horizontal scale shows fragment size, from 80 to 570 bp (left to right).. Each time is represented by three replicates. B) NMDS diagram for the same experiment showing samples kept at 4°C (numbers, following increasing times from 15 min to 4 hours) or at 19°C (letters, following alphabetical order from 15 min to 4 hours). Times correspond to 15, 30 min, 1, 2, or 4 hours.

Differences with time were tested again with a culture of *Micromonas pusilla* (experiment 3). Triplicates were done for each sampling point, from 15 min to 4 h, maintaining replicates of *M. pusilla* cultures at 4°C or at 19°C. Sampling and filtering were practically instantaneous, with no time delay. The primer combination A8 was used to obtain the fingerprints (Figure 4A) and distances among them were represented in a NMDS diagram (Figure 4B). No substantial differences were observed from 15 min to 2 h in samples kept at 4°C (except for one of the triplicates from 2 h that was an outlier). Slightly larger differences were observed at 19°C, even though all the samples were more than 70% similar to the t = 0 ones. At 4 h, however, both samples 4°C and at 19°C were significantly different from t = 0. In addition, the triplicates of samples kept at 19°C were very different from each other.

We carried out permutational multiple analysis of variance (PERMANOVA) with the results from experiments 2 and 3. In both cases, time resulted in the largest differences among samples (r2 = 0.455 and 0.495 respectively, p = 0.001 in both experiments). Temperature was also significant in both experiments although it explained a lower percentage of variability than time (r2 = 0.232 and 0.066 respectively, p = 0.001 and 0.02).

There were too few replicates in experiment 2 to carry out ANOSIM pairwise tests, but in experiment 3, the R values gradually increased between 15 min and 2 h for samples in ice, while they were high already at 15 min at room temperature. In conclusion, as could be expected, time should be kept as short as practical, but keeping the samples in ice will decrease the problem.

### *Micromonas pusilla* gene expression under dark and light conditions

**Figure 5:**
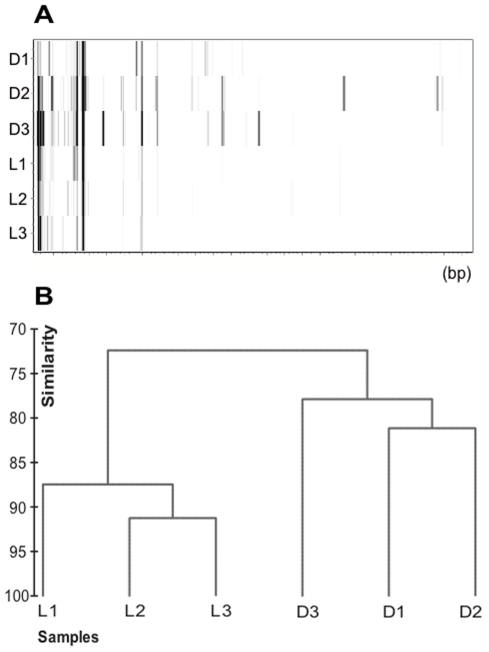
*Micromonas pusilla* gene expression under dark and light conditions. A) Virtual gel of the TFA patterns for experiment 4. The horizontal scale shows fragment size, from 80 to 570 bp (left to right). D: dark samples, L: light samples. Numbers are replicates for each condition. B) Dendrogram showing the clustering of light and dark samples for the same experiment.

In experiment 4, a culture of *Micromonas pusilla* was incubated both in the dark and in the light. Different TFA patterns were observed (Figure 5). With the primer combination used, the number of cDNAs retrieved in dark samples was larger than in light samples, but the total number of peaks was very small so that the significance of the differences was low (Figure 5). This particular example shows how the technique can also be used to identify genes with differential regulation under the experimental conditions tested (as is the case in differential display).

### Relationship of TFA patterns with different primer combinations

**Figure 6:**
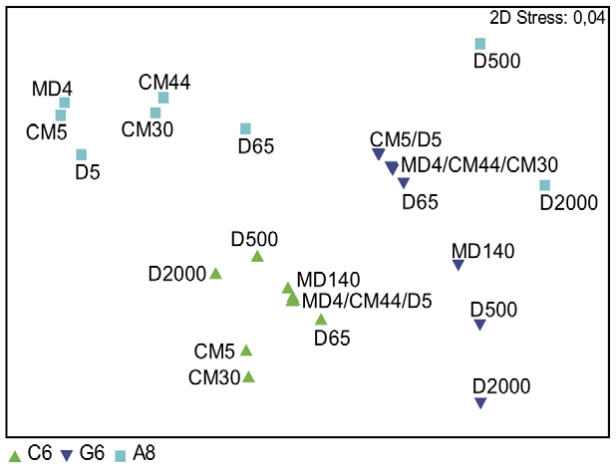
Relationship of TFA patterns with different primer combinations. NMDS diagram comparing fingerprints obtained with three different primer combinations from the MODIVUS transect from the coast to offshore in the NW Mediterranean Sea. Samples are labeled according to station (CM, MD, and D) and depth (4 to 2000 m). The number added after the name of the station indicates depth. The primer combinations used were A8, C6 and G6. Sample MD140 analyzed with the A8 primer combination was very distant from all the others and has not been represented for clarity.

To check whether different primer combinations would cluster samples similarly, samples from three vertical profiles (from stations CM, MD and D) were selected (see location of samples in Table 3) and the procedure was run with three different primer combinations (A8, C6 and G6). The NMDS diagram in Figure 6 presents the ordination of TFAs from all these runs. TFAs obtained with A8 showed that samples separated along the depth gradient, the largest distances appearing between intermediate and deep samples. Near-surface samples obtained with the A8 primer combination clustered together. With the C6 combination deep samples were also separated from the rest of the samples but the distance between all the samples was much less compared to the separation obtained with the other combinations. The G6 combination also resolved the vertical profile but the intermediate depths were not as well separated as with the A8 primer combination.

**Table 3:** Location and depths of samples analyzed in Figure 6.

Station	Latitude N	Longitude E	Depths sampled (m)
CM	41°24′	2°48′	5/30/44
MD	40°54′	2°50′	4/140
D	40°39′	2°51′	5/65/500/2000

Altogether the surface samples of the horizontal transect clustered together for each primer set and largest differences were observed along the depth profile. This was very clear with primer combinations A8 and G6. In contrast, the C6 primer combination was not as good at resolving the vertical gradient. The A8 primer combination was chosen for all our analyses for its resolution and repeatability.

## Discussion

The aim of this study was to develop a fingerprinting method that could track changes in microbial community gene expression patterns and that was compatible with usual working conditions in oceanographic cruises. The main challenge in a cruise is to obtain sufficient mRNA in as short time as possible. First, samples from several thousand meters deep may take several hours to reach the lab on board. The ideal solution would be to fix the samples at *in situ* depth. However, there is no commercially available sampling bottle able to do this. Besides, fixing *in situ* requires large amounts of fixative making the whole operation impractical and environmentally harmful. And, second, open sea oligotrophic waters have very low concentration of microorganisms and require more filtration time. One possibility is to use mRNA amplification methods. However, these add an additional step that makes the procedure more expensive and complex. We wanted to test whether we could find a method that would provide representative gene expression patterns for a large number of samples despite these difficulties. We chose differential display and modified this technique for faster and easier processing. This technique was developed primarily to identify genes expressed in tumor cells versus normal cells [27]–[29]. The technique is simple as it is based on PCR and conventional sequencers, it is sensitive and repeatable, and relatively quick and economical. Moreover, TFA does not require prior knowledge of the mRNA sequences to be detected. This last characteristic is especially important for the study of natural communities.

Of course, there are several common difficulties and assumptions when differential display is used. First, a band in gel electrophoresis (or a peak in our case) might be due to several genes. And, conversely, one gene could be represented by more than one fragment. This is also the case with DNA fingerprinting techniques such as DGGE, T-RFLP, or ARISA. Particularly in a mixed natural community, gene fragments of identical length could originate from different microorganisms. As long as these events are repeatable, however, they are of no concern for the fingerprinting objective.

A second concern is the potential bias of the reverse transcription. It is well known that the experimental variation in a RT-PCR process is mainly attributable to the reverse transcription step [30], [31]: although PCR is a cyclic reaction that accumulates errors, its repeatability is significantly higher than that of the single-step reverse transcription reaction [30], where there are several factors that could influence the final product. In order to improve the repeatability of the assay we optimized the process by testing several RT enzymes and annealing temperatures. With the thermostable reverse transcriptase chosen we obtained highly repeatable peaks in repeated reactions, not only for the large peaks but also for the small ones (Figure 2). The high annealing temperature during reverse transcription reduced the degree of mRNA secondary structure, which is substantial in the 3′untranslated region (3′UTR) we were targeting by using of oligo(dT) primers. In addition, the RT might preferentially amplify some mRNAs, thus altering the relative proportions of the genes being expressed. In particular, shorter mRNAs might be preferentially used as targets [32]. We did not find any significant differences in this respect when we tested different reaction times or when we compared peaks corresponding to different sizes.

And third, the final PCR step is subject to the usual PCR biases, and some cDNAs might be differentially amplified. As a result of the two latter caveats, the relative proportions of the expressed genes in the final fingerprint may not be exactly as they were in the natural sample. Because of the clearly delimited purpose of the approach, however, this would not be a problem either as long as the biases were repeatable.

As shown in the Results section, the expression patterns found were always highly repeatable under the conditions used. Moreover, for the approach to be useful as a fingerprint it was not necessary to be able to identify the genes being expressed, to determine how many genes were being expressed at a particular moment, or to quantify the expression of the different genes.

Here, TFA was developed and applied to picoeukaryotic communities taking advantage of the poly(A) tail of mRNA in eukaryotes. However, TFA can be easily modified to be used with prokaryotes by previously removing rRNA and subsequent polyadenilation of the RNA of the bacterial fraction as described in [1].

According to the manufacturer of the kit (www.genhunter.com), the use of three oligo(dT) primers (for the reverse transcription reaction) plus eighty random primers (for the subsequent PCR) will retrieve 96% of the genes in any given eukaryotic cell. Since only one of three oligo(dT)s and only one of eighty random primers were used in the present work, the fingerprints corresponded to a very small fraction of all the genes being expressed at any one time. In effect, when the technique was applied to a pure culture of *M. pusilla* the number of peaks was very low in one of the experiments (Figure 5). This number of peaks would not be enough for a proper classification of samples. However, when the same technique was used with natural samples, in which a mixture of cell populations is present, the number of peaks was sufficient. Economy of resources and reactions being essential for a convenient fingerprinting technique, we decided that the use of one random primer and one anchor primer was the most efficient alternative.

Since the primer combinations are arbitrary, the transcripts retrieved with each set should be a random representation of the genes being expressed at the time of sampling. Therefore, most primer combinations should result in similar clustering of samples. However, the resulting clustering will be more robust if there are more peaks and there is a range of peak heights. Since this will change at random with the primer sets and the particular communities being analyzed, optimization requires testing different primer combinations for each type of environment studied. In the case of the Mediterranean waters analyzed the primer combination A8 was the best at discriminating samples from the vertical profile (Figure 6) and was, thus, chosen as our preferential combination for subsequent reactions. Likely, the primer combination will have to be optimized for each type of sampling. Once this has been done, the procedure is relatively cheap and quick.

In order to have a positive control, replicate cultures of *Micromonas pusilla* were incubated in the light and in the dark. It is well known that transcripts of algae change dramatically between day and night [33]. Obviously, if the technique is to work in nature it should be able to detect differences between light and dark incubations in a phototrophic protist. The patterns were clearly different, revealing more transcriptional activity in the dark than in the light with the primer combination used. This can be expected since phototrophs tend to concentrate on carrying out photosynthesis during the light hours, while the dark is used for biosynthesis of all the different cell components plus all the regulation involved in nucleus and cell division. As mentioned earlier, the TFA is proposed here only as a fingerprinting technique. Despite this, in some cases it may be of interest to identify some of the genes observed. If a gene turned out to be relevant, the sample could be run in a polyacrylamide gel and the corresponding band could be cloned and sequenced.

The main challenge in a cruise is to obtain sufficient mRNA in as short a time as possible to prevent major changes in the transcript composition from the fresh sample (this is due to the labile nature and relatively short half-lives of mRNAs). Unfortunately this is not always possible: as explained, samples from lower depths take hours to reach the lab on board, and oligotrophic waters have very little material and require more filtering time. Therefore, another important concern was to assess to which extent the time delay between sampling and filtering affected gene expression. The two timing experiments with natural marine communities supported the idea that time did not significantly alter the patterns of gene expression as long as samples were kept on ice, for the picoeukaryotic transcripts retrieved at least up to two hours after sampling with several specific primer combinations.

In summary, TFA is a compromise among the different requirements that provides a repeatable gene expression pattern in a relatively simple and inexpensive way and that will be practical to use in oceanographic cruises. Results suggest that TFA is a useful technique when a large number of conditions or treatments have to be compared side by side, by assessing a portion of the genes expressed by such communities. TFA is an indicator of the extent of changes caused by different environmental conditions. This previous analysis would then help in deciding which samples to use for more powerful, but time-intensive (and costly) methods for estimating gene expression patterns.
